# EBM BLS: Zoledronate Reduces Vertebral Fracture Risk in Young Postmenopausal Women Without Osteoporosis

**DOI:** 10.1007/s11606-026-10415-5

**Published:** 2026-04-27

**Authors:** Christian Hannah, Jason T. Alexander, Samuel W. Trump

**Affiliations:** 1 Department of Medicine, University of Chicago, Chicago, IL USA; 2 Department of Medicine, University of Wisconsin School of Medicine and Public Health, Madison, WI USA

**Keywords:** zoledronate, fractures, postmenopausal women

**Source Article:** Bolland MJ, Nisa Z, Mellar A, et al. Fracture Prevention with Infrequent Zoledronate in Women 50 to 60 Years of Age. N Engl J Med. 2025 Jan 16;392(3):239-248. https://doi.org/10.1056/NEJMoa2407031.

## WHY THIS IS IMPORTANT

- Approximately 70% of fragility fractures in women occur in patients without osteoporosis [1], representing a population of patients where additional therapeutic guidance to decrease fracture risk is needed.
- Studies evaluating therapies to reduce fractures among women without osteoporosis have been inconsistent [2].
- Zoledronic acid is used to prevent fractures and is administered every 12 to 18 months, and its effects on bone density and turnover have been shown to persist beyond 5 years [3].

## INTERVENTION

- Women participants (n = 1,054) were assigned in a 1:1:1 ratio to receive either an intravenous infusion of zoledronate 5 mg at baseline and 5 years (zoledronate-zoledronate); zoledronate 5 mg at baseline and placebo at 5 years (zoledronate-placebo); or placebo at baseline and 5 years (placebo-placebo).
- No additional interventions such as vitamin D or calcium supplementation or patient education was offered as part of the trial.
- Participants were evaluated for fractures and adverse events every 6 months via patient reported questionnaires, and radiographs were performed at baseline, 5, and 10 years.
- The primary outcome was new morphometric vertebral fracture as determined by spinal radiograph.
- Overall, 95.2% of participants completed 10 years of follow up.

**Figure 1:**
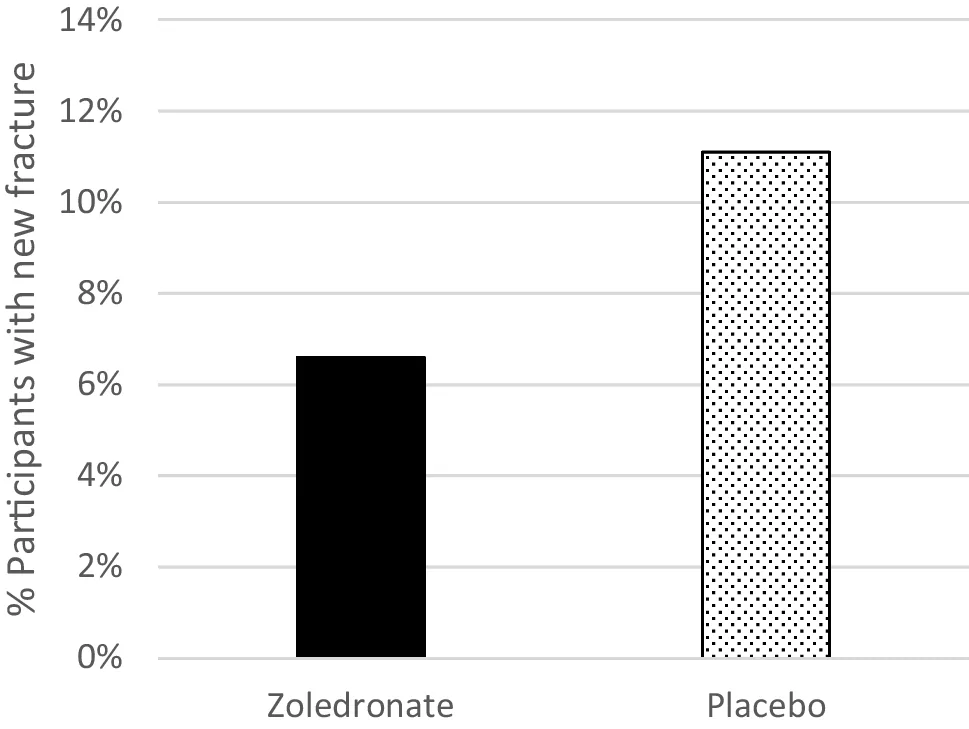
Comparison of new vertebral fracture rates over 10 years.

## RESULTS

- Mean age was 56.0 years, 84.9% self-reported European ancestry, average lumbar spine T-score was -0.4, and average total hip T-score was -0.5.
- The primary outcome occurred in 6.6% in the zoledronate-placebo group (relative risk (RR) = 0.59, 95% CI 0.36 to 0.97, p = 0.08 compared to placebo-placebo group), and 11.1% in the placebo–placebo group (Fig. 1). An additional dose of zoledronate at 5 years was similar to one dose (RR = 0.56, 95% CI 0.34 to 0.92).
- The number needed to treat to prevent one woman from having a new morphometric vertebral fracture during the 10-year period was 22 in the zoledronate–placebo group.
- Both zoledronate treatment groups showed higher lumbar spine and total hip bone density than the placebo group at 5 and 10 years.
- Lower bone turnover markers were also noted in the zoledronate treatment groups by about 30–40% as compared to placebo.
- Adverse event rates were low in all treatment groups, although 8 cases of uveitis and 1 case of episcleritis were reported in the treatment groups receiving at least one dose of zoledronate, with none reported in the placebo-placebo group.
- No cases of atypical femur fracture or osteonecrosis of the jaw were reported.

## STUDY DESCRIPTION

### Setting

- Double-blind, randomized, prospective, placebo-controlled trial conducted at a single center in New Zealand from 2012 to 2023.
- Participants were postmenopausal women 50 to 60 years of age with a bone mineral density T-score at the lumbar spine, femoral neck, or total hip < 0 and > -2.5.
- Intention-to-treat analysis was used.

### Exclusion Criteria

- T-score ≤ -2.5, any major systemic illness or metabolic bone disease, a previous clinical spine or hip fracture, use of any bisphosphonates or hormone-replacement therapy within 12 months before randomization, any previous use of zoledronate, or use of oral glucocorticoid drugs equivalent to an average dose of prednisone of 2.5 mg per day or more during the preceding 6 months.

## STUDY QUALITY AND APPLICATION TO PATIENTS

- The study quality is good.
- Strengths include its clinically relevant design, long follow-up, and high participant retention.
- Limitations include it was single center and had low racial and ethnic diversity which somewhat limits its generalizability.
- Implementation of these results into current practice in the US is limited by the lack of insurance coverage of DEXA tests in women prior to 65 without certain risk factors and elevated fracture risk [4].
- Whether other routes of zoledronate administration (ie, subcutaneous injection) would confer similar benefit to patients is unknown.

## Data Availability

N/A.
